# Digital monitoring of motor function in Parkinson’s disease using Markerless motion analysis and exergaming

**DOI:** 10.3389/fneur.2026.1800332

**Published:** 2026-04-15

**Authors:** Claudia Ferraris, Gianluca Amprimo, Guido Coppo, Francesca Bellanova, Matteo Bigoni, Alessandro Mauro, Lorenzo Priano

**Affiliations:** 1 Institute of Electronics, Computer and Telecommunication Engineering, National Research Council, Turin, Italy; 2 Department of Control and Computer Engineering, Politecnico di Torino, Turin, Italy; 3 Synarea Consultants S.R.L., Turin, Italy; 4 Division of Neurology and Neurorehabilitation, S. Giuseppe Hospital, IRCCS Istituto Auxologico Italiano, Piancavallo (VB), Italy; 5 Department of Neurosciences “Rita Levi Montalcini”, University of Turin, Turin, Italy

**Keywords:** AI-assisted rehabilitation, digital medicine, exergaming, Google MediaPipe, markerless body tracking, motion analysis, Parkinson’s disease

## Abstract

**Introduction:**

Motor impairment in Parkinson’s disease (PD) significantly compromises functional independence. While continuous rehabilitation is crucial, traditional models face logistical and economic barriers that limit continuity of treatments.

**Methods:**

To address this challenge, we developed a novel exergaming platform leveraging Google MediaPipe for markerless, real-time kinematic tracking via a standard webcam, eliminating the need for specialized hardware and delivering engaging, gamified physical exercises designed for domestic settings. This study investigates the feasibility, usability, and preliminary clinical impact of a 10-session gaming protocol in 14 out-of-hospital patients.

**Results:**

The system showed high technical performance and participant engagement, with an overall trial completion rate exceeding 94% and successful progression through game levels. We observed improvements in key functional parameters, establishing a strong correlation between level progression, measured by the novel Normalized Efficiency Index, and the clinical MDS-UPDRS assessments, both for total (*ρ* = −0.61) and mobility (*ρ* = −0.66) scores. Furthermore, the system detected performance incongruence related to medication timing and motor fluctuations. In addition, a session-by-session analysis revealed consistently high patient satisfaction, engagement, and system usability scores, alongside low perceived physical fatigue.

**Discussion:**

These findings underscore the clinical validity and high acceptance of the proposed solution as a training and remote monitoring tool. By providing granular, longitudinal data, this highly accessible solution offers a promising approach to personalized home-based functional training for people with PD.

## 1 Introduction

One of the core challenges in managing Parkinson’s disease (PD) lies in the progressive deterioration of motor skills, directly resulting from the loss of dopaminergic neurons (1). This neurobiological deficit manifests as debilitating and typical motor symptoms (e.g., bradykinesia, rigidity, tremor, and axial stiffness), which collectively lead to reduced joint mobility, poor motor control and coordination, and compromised execution of goal-directed movements (2, 3). Consequently, patients struggle with complex or asymmetric bimanual tasks (4) and with maintaining postural control, which sharply diminishes functional autonomy and increases the risk of falls (5). Furthermore, the decline in motor function is not isolated, but it is strongly associated with non-motor complications, such as depression, social isolation, and cognitive decline, compounding the overall clinical burden (6–9).

Sustained physical intervention and targeted motor training are recognized as cornerstones of non-pharmacological PD management, demonstrating efficacy in preserving functional abilities, improving balance, and potentially inducing beneficial neuroplasticity (10–12). The clinical evidence is substantial: systematic reviews confirm that constant and frequent physical activity helps maintain motor function and balance across different stages of the disease (13). In addition, home-based and personalized physical interventions show significant promise in enhancing mobility and functional performance, especially for individuals in the early to mid-stages of PD. (14)

However, the efficacy of exercises is critically dependent on their continuity, frequency, and personalization. Traditional clinic-based physiotherapy models frequently fail to meet these requirements due to persistent economic, logistical, and geographical barriers (15, 16). These constraints often preclude the high-frequency and long-term adherence necessary for effective treatment of a chronic, progressive condition like PD. In addition, the efficacy of traditional physiotherapy is often limited by its episodic nature: clinic-based treatments provide only temporary snapshots of the patient’s condition, which may be influenced by pharmacological fluctuations and fail to capture the patient’s daily functional performance. Addressing this crucial, unmet need requires a paradigm shift toward accessible, home-based therapeutic solutions that support continuous, engaging, and personalized motor practice (17).

Recent research has pivoted toward leveraging Artificial Intelligence (AI) and advanced digital technologies to enable remote, personalized motor training (17). A key technological enabler of this transition is Computer Vision, particularly thanks to innovative Human Pose Estimation (HPE) algorithms (18–20). HPE approaches facilitate the analysis of kinematics and movement quality directly from standard cameras, such as webcams or smartphones. This effectively eliminates the need for complex commercial optical devices (such as RGB-D sensors), wearable sensors, or marker-based systems (21, 22). This capability is crucial for developing solutions and designing interventions that are highly scalable, cost-effective, and non-invasive, making them ideal for implementation in patients’ homes.

Significant advancements in this area are exemplified by the Google MediaPipe Pose (GMP) framework (23). Utilizing lightweight, optimized neural networks, GMP enables accurate, real-time skeletal tracking using only ubiquitous standard RGB devices. This approach offers a critical technical advantage: it maintains sufficient precision for real-time movement analysis while substantially reducing the complexity and expense associated with hardware-intensive solutions (24–27). Moreover, transitioning to more widely available standard cameras allows us to go beyond the limited lifecycles and rapid obsolescence of RGB-D devices that have been observed in recent years (28).

The high accessibility of GMP technology makes it an ideal fit for integration into exergames, which are interactive games specifically designed to promote exercise and physical activity (29, 30). Exergames capitalize on the motivational appeal of video games to enhance treatment adherence and compliance, a factor often problematic in long-term rehabilitation (31–33). Scientific evidence supports this methodology, showing that gamified tasks yield multifaceted benefits: they demonstrate to improve physical outcomes (e.g., balance, coordination, and reaction time) while simultaneously boosting cognitive and emotional domains, including motivation, enjoyment, and perceived self-efficacy (34–39).

Within this context, we evaluate a novel, markerless, 3D exergame platform, developed for motor training in PD, using GMP body tracking via standard webcam input. The platform is designed specifically to prioritize non-invasiveness, accessibility, intuitiveness, and user engagement, supporting the continuous practice of exercises in a home environment. This pilot study pursues two main objectives: first, to evaluate the operational feasibility and acceptability of a GMP-based exergame protocol during a 10-session intervention in a community-based setting, and second, to demonstrate the system’s capability to provide objective functional metrics that can track individual motor performance and detect subtle motor fluctuations over time. In addition, the study introduces the Normalized Efficiency Index (NEI), a dedicated metric designed to quantify performance efficiency by balancing task difficulty with execution quality, and evaluates its correlation with standard clinical scales.

The preliminary results of this pilot study offer valuable insights regarding the sustainability, motivational potential, and clinical usefulness of such technologies for long-term motor rehabilitation. By doing so, the study aims to demonstrate that digital exergaming can transcend its role as a simple training tool to become a reliable remote monitoring instrument capable of detecting changes in the patient’s functional performance that traditional clinical snapshot assessments might miss.

## 2 Materials and methods

### 2.1 Participants

A cohort of 25 volunteers was recruited from a local Parkinson’s disease (PD) patient association for an experimental data collection phase conducted from March to May 2025 as part of the ELEVATOR project. All procedures were performed in accordance with the Declaration of Helsinki and received formal approval from the local Ethics Committee (Protocol No. 474/2024, issued on April 4, 2024). Before enrollment, all participants provided written informed consent after receiving a detailed explanation of the study’s objectives and the nature of the exergaming activities.

One week before starting the gaming protocol, a neurologist with extensive experience in movement disorders conducted a thorough baseline assessment (T0) to characterize the motor profile and clinical condition of each participant. In accordance with the study protocol, no further clinical assessments were scheduled, since the primary focus was on evaluating game-derived digital metrics. The baseline assessment was based on the Hoehn & Yahr (H&Y) scale (40) and the motor examination (Part III) of the Movement Disorder Society-Unified Parkinson’s Disease Rating Scale (MDS-UPDRS) (41). The MDS-UPDRS tasks were evaluated to produce the total motor examination score (UPDRSIII_SCORE_) and to define specific sub-scores: the limb mobility score (MOBILITY_SCORE_), calculated by summing items 3.4–3.7; the gait and posture score (GAITPOS_SCORE_), by summing items 3.9–3.13; the bradykinesia score (BRADY_SCORE_), based on item 3.14; the tremor score (TREMOR_SCORE_), by summing items 3.15–3.18; and the rigidity score (RIGIDITY_SCORE_), obtained by summing the scores for the neck, arms, and legs from item 3.3.

Out of the initial 25 volunteers, 15 met the inclusion criteria. Eligibility was based on the following requirements: a confirmed diagnosis of idiopathic Parkinson’s disease (PD) according to the Movement Disorder Society (MDS) clinical diagnostic criteria (42); an overall clinical condition allowing for safe and appropriate interaction with the technology; and the potential to benefit from exergame-based motor training. Cognitive impairment, assessed through neurological screening at baseline, was an exclusion criterion to ensure comprehension of the game instructions and the effective use of the computer-based solution. Participants with comorbidities preventing system use were also excluded. The demographic information and baseline clinical characteristics of the participants are reported in Table 1.

**Table 1:** Demographic information and baseline (T0) clinical assessment of the initially enrolled participants (*n* = 15) and the final analyzed cohort (*n* = 14).

Demographic information	Initially enrolled (*n* = 15)	Final cohort (*n* = 14)
Gender	12 Males/3 Females	12 Males/2 Females
Age (years)	71.80 ± 5.65 [54–81]	72.11 ± 5.90 [54–81]
Years from diagnosis	7.27 ± 6.25 [3–15]	7.86 ± 4.70 [3–15]
Baseline clinical assessment^1^		
H&Y_SCORE_	2.46 ± 0.66 [2–4]	2.46 ± 0.66 [2–4]
UPDRSIII_SCORE_	26.00 ± 13.26 [14–62]	26.50 ± 13.61 [14–62]
MOBILITY_SCORE_	10.83 ± 6.61	10.75 ± 6.85
GAITPOS_SCORE_	3.73 ± 2.32	3.79 ± 2.40
BRADY_SCORE_	1.60 ± 0.99	1.64 ± 1.01
TREMOR_SCORE_	2.77 ± 2.31	2.96 ± 2.27
RIGIDITY_SCORE_	3.03 ± 3.63	3.25 ± 3.65

^1^All clinical scores are expressed in points (pts). The final cohort includes all subjects who successfully completed the gaming protocol. Data are presented as mean ± standard deviation. Range [(min – max)] is reported where relevant.

### 2.2 Experimental gaming protocol

Although the exergaming platform is intended for unsupervised home use, all gaming sessions for this preliminary study took place at the local patient association facility where the participants were recruited. The gaming sessions were conducted under the supervision of trained operators. The experiment included an initial familiarization session conducted concurrently with the baseline clinical motor assessment, followed by a multi-session gaming protocol spanning up to 4 weeks. The protocol was designed to be flexible in order to accommodate participants’ logistical needs and travel availability to the association’s facility. The intervention consisted of a maximum of 10 gaming sessions per participant, scheduled one to three times per week over a four-week period. Each gaming session lasted approximately 45–60 min, depending on the number of attempts (trials) and the participant’s perceived fatigue. During a single session, participants performed all four exergames (Airplane, Ski, Piano, and Gym, as described in Section 2.3) in a fixed sequence. This non-randomized approach was intentionally adopted to ensure a consistent performance baseline across participants and sessions, thereby minimizing intra-subject variability and enabling direct comparisons of progress within each task. To prevent overexertion, rest breaks could be taken at any time at the participant’s request.

In line with the study’s primary objectives and to ensure a continuous therapeutic challenge, all participants started at the baseline level (Level 0) for each exergame in the first gaming session. Game difficulty increased progressively based on successfully achieving exergame-specific objectives (e.g., completing a level without errors), either within a single session (in the case of multiple intra-session exergame trials) or across subsequent sessions. In all subsequent gaming sessions, participants resumed from the highest difficulty level they had successfully reached for each exergame in their previous session, without restarting from Level 0. To mitigate the risk of excessive fatigue, a critical factor in PD, the number of trials per session was strictly regulated according to the level of physical exertion demanded by each specific exergame and the patient’s condition on the session day.

### 2.3 Exergame design and clinical rationale

The exergames presented in this study evolved from versions originally developed for RGB-D sensors, specifically the Microsoft Azure Kinect (25, 43, 44). Within the current framework, these games have been significantly redesigned and refactored to ensure compatibility with standard RGB webcams via the Google MediaPipe Pose (GMP) framework (23), improve accessibility, and simplify hardware requirements. Furthermore, the exergames have been organized into a unified *web-based platform* that manages game activation on a *local system*, centralized data collection, and dedicated dashboards for clinicians.

The exergames were specifically designed to stimulate body movements and target key motor functions affected by PD symptoms. The exercises reproduce some strengthening and mobility tasks commonly included in traditional rehabilitation protocols to train and improve motor skills, as highly recommended by physiotherapists for PD management (45–47). The suite included the following exergames:

- *Airplane*: This game focuses on trunk postural control and mobility through mediolateral and backward-forward movements. Further details on game design are provided in Supplementary Table S1. Since the required physical exertion is moderate and of short duration, the protocol allowed for a maximum of three trials per session.
- *Ski*: This game focuses on lower-limb mobility and coordination through rhythmic alternating leg movements, combined with occasional upper-limb movements to adjust the avatar’s trajectory. More details are available in Supplementary Table S2. Given the high intensity and complexity of the movements, the protocol limited this exergame to a maximum of two trials per session.
- *Piano*: This game focuses on arm-pointing precision through vertical and lateral movements. Details are provided in Supplementary Table S3. As the required effort is moderate and of short duration, up to three trials per session were permitted.
- *Gym*: This game focuses on the rhythmic repetition of frontal and lateral upper-limb raises in various modes (single-arm, simultaneous, or alternated). More details can be found in Supplementary Table S4. As the game consists of eight distinct exercises (four frontal and four lateral), the protocol allowed for only one complete trial per session.

As described in Supplementary materials, each exergame features a progressive difficulty structure with trials distributed across highly granular levels. This multi-level design was specifically adopted to avoid abrupt difficulty spikes that could frustrate PD patients, enabling precise micro-tailoring of progression according to each patient’s motor capabilities. Game progression relies on the combined configuration of various game parameters and, in some cases, the introduction of new motor and cognitive demands. In Airplane and Ski, difficulty increases with greater trajectory complexity and faster game speeds. For example, increasing the avatar’s speed requires faster motor responses, while extending the track length demands prolonged motor execution. At higher levels, these games also introduce novel motor or cognitive demands. For example, the Airplane game incorporates a take-off phase that engages trunk movements alongside cognitive challenges, such as flying through the correct color-coded circles. In contrast, the Ski game introduces curved tracks that require highly coordinated arm movements. In the Gym game, progression is defined by the number of arm-raising repetitions, and in Piano, difficulty increases with the length of the key-press sequence. While no new movement types are introduced in these two exergames, the longer sequences progressively challenge the participant’s endurance, by placing sustained demands on motor control and coordination to engage these motor domains. It is important to note that while the specific number of levels used in this preliminary study was adapted to our current protocol, the underlying exergaming platform is inherently parametric. The games are designed to be fully customizable, allowing new intermediate levels to be introduced flexibly, if necessary, to adhere even more precisely to patients’ evolving motor performance and clinical needs.

### 2.4 Local system architecture and motion tracking

The local system was designed to provide a sustainable, scalable tool that moves beyond the limitations of specialized hardware by leveraging innovative markerless motion-tracking frameworks operating on standard RGB video streams. This transition was strategically planned to ensure long-term technical sustainability and to facilitate the future integration of new exergames, particularly for widespread use in home-based applications.

**Figure 1:**
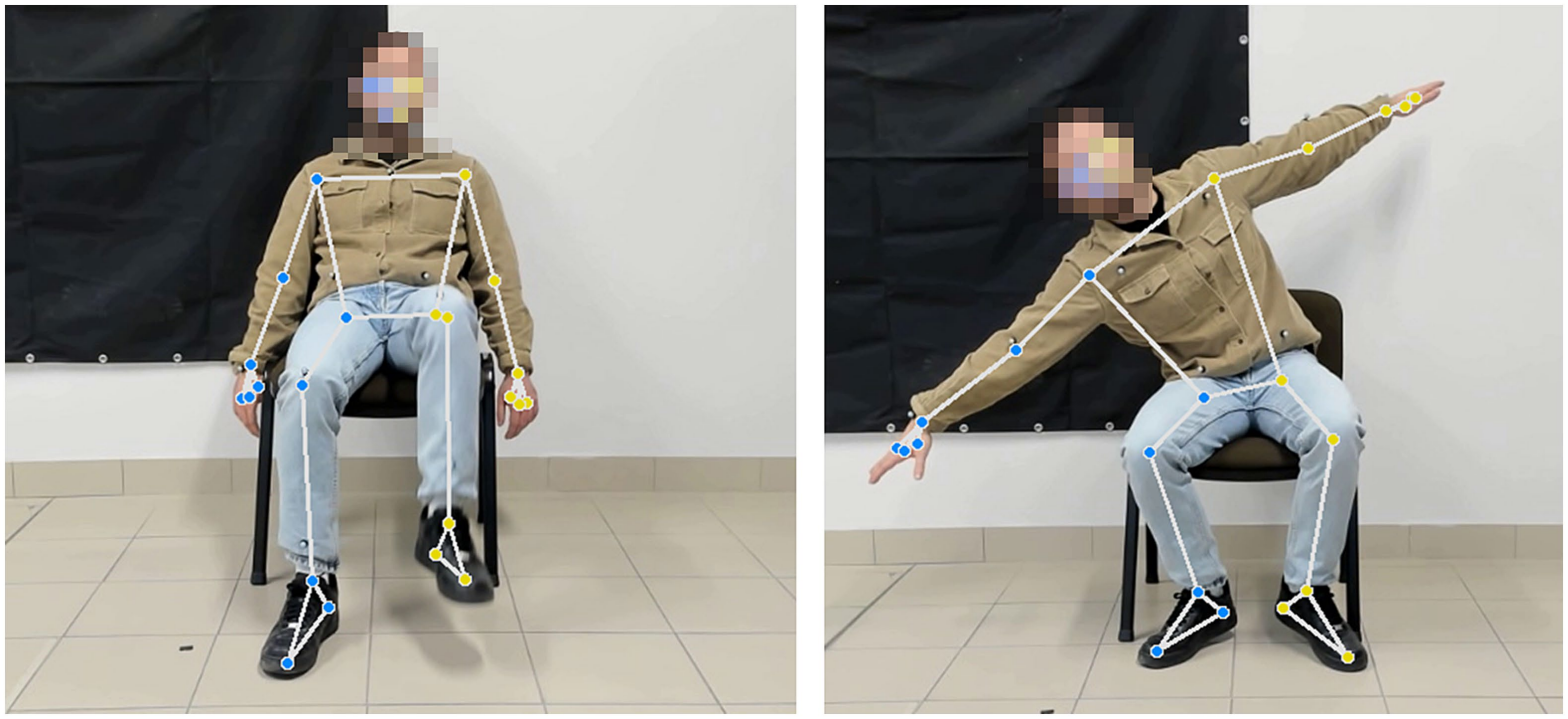
Example of the GMP markerless motion tracking. The 2D skeletal model estimated by the GMP framework is superimposed on a frame captured during the SKI and AIRPLANE exergames. The identified landmarks, converted into 3D world coordinates, enable real-time tracking of trunk, upper-limb, and lower-limb movements.

The architecture follows a modular approach consisting of two main components: the Motion Tracking Module (MTM), developed in Python v0.10.21, which manages the camera’s video stream and implements the GMP framework for pose estimation; and the Virtual Interaction Module (VIM), developed in Unity, which handles the game logic, user interface, and 3D rendering of the exergame scenarios. To balance high-fidelity landmark detection with computational demands, the GMP framework was configured with “Full” model complexity (28) and a confidence threshold of 0.7. The MTM estimates a 2D skeletal model of 33 landmarks in image coordinates, which is subsequently uplifted into a 3D skeletal reconstruction in world coordinates. This dimensional uplifting is crucial for the objective characterization of 3D movements and the extraction of reliable kinematic parameters. Figure 1 shows a representative example of the 2D skeletal landmarks estimated by the GMP framework.

The two modules exchange data via a WebSocket interface, which continuously transmits the 3D coordinates of the 33 landmarks estimated for each RGB frame from the MTM to the VIM. This pipeline enables the real-time reconstruction of the user’s movements within the virtual environment. To ensure a smooth, responsive experience, the system was deployed on a laptop (Intel Core i7-9750H, 16 GB RAM, NVIDIA GTX 1650), achieving 30 frames per second at 720p resolution.

Finally, the kinematic data and game results are stored locally as JSON files and transferred to the platform’s centralized data management layer for offline analysis and longitudinal clinical monitoring. To comply with privacy-by-design principles, data are transmitted and stored in an anonymized format on a secure cloud server, ensuring that participants’ confidentiality is fully maintained. Data access is managed through a multi-level authentication system to ensure that only authorized clinical personnel can review participants’ longitudinal progress.

### 2.5 Data processing and performance analysis

Objective characterization of motor performance was derived from 3D trajectories of skeletal landmarks recorded during each trial. Data analysis was performed using MATLAB (v.2024a, MathWorks, Natick, MA, United States) through custom-developed scripts tailored to each exergame.

#### 2.5.1 Signal pre-processing

To ensure high data quality, the 3D landmark trajectories underwent a standardized pre-processing routine. First, a median filter with a window size of 8 samples was applied to each coordinate component (X, Y, and Z) to eliminate artifacts, spurious spikes, and tracking outliers. Median filtering is highly effective at removing impulsive noise while preserving signal edges, movement shape, and dynamics, and it does not introduce distortions, especially when short windows are used (48). Next, the trajectory components were resampled at 50 Hz using cubic spline interpolation to increase spatial resolution and standardize the time base across trials. This is a common practice in human movement analysis applications that use standard cameras at 30 frames per second (49). This step was introduced to mitigate the jitter associated with vision-based acquisition systems and the uncertainties of markerless body tracking algorithms when estimating anatomical landmark positions (50). Although resampling is primarily used to compare different tracking systems (e.g., during validation with gold-standard systems), upsampling was employed here to obtain a more robust, uniformly sampled time series and to ensure the stability and reliability of the derived kinematic metrics (51). Finally, a third-order low-pass Butterworth filter was applied with a cut-off frequency of 10 Hz. This filter is one of the most widely adopted in biomechanical analysis (52) as it removes high-frequency noise, ensuring smooth trajectories for subsequent kinematic analysis, while preserving the kinematic characteristics of the trajectory components (53, 54). Importantly, the selected cut-off frequency retains the frequency band of voluntary movement (55, 56), while almost completely removing tremor interferences (> 7.5 Hz), which fall outside the scope of this study.

#### 2.5.2 Motor and game-based features

Following the pre-processing stage, two distinct sets of features were extracted: motor functional parameters (MFP) and game-based performance metrics (GBM). A complete list of all MFPs and GBMs for each exergame is provided in the Supplementary Table S5.

MFPs were derived directly from the 3D trajectories to objectively quantify movement quality. Since each exergame targets different body segments, specific parameters were defined to capture physical quantities characterizing body movements, including joint angles, velocities, and range of motion (ROM). Given the characteristic lateralization of symptoms in Parkinson’s disease, a Symmetry Index (SI) was calculated for bilateral parameters using Equation (1):


PSI=∣(PRPL)−1∣ (1)

Where P represents a generic functional parameter, while P_R_ and P_L_ denote the values recorded for the right and left sides of the body, respectively. According to this metric, a value close to 0 indicates almost perfect bilateral symmetry, while values deviating from 0 indicate an increasing degree of asymmetry, providing an objective measure of the functional gap between the more and less affected sides.

Angular measures ( θ ) were computed to evaluate postural and limb orientation using the dot product between two body segments (vectors v_1_ and v_2_), defined by pairs of 3D landmarks, according to Equation (2):


$$\theta =\arccos\;(\frac{v_{1}.v_{2}}{\left\|v_{1}\|\|v_{2}\right\|})$$ (2)

GBMs were automatically recorded by the Unity-based VIM to assess the ability to achieve task-specific goals, including total completion time, average number of points, errors, and pauses per trial. The integration of MFPs and GBMs aims to provide a comprehensive performance profile that captures both the kinematic quality of the movement and the functional success in the gamified environment.

### 2.6 Feasibility, usability, and engagement assessment

To evaluate the proposed solution systematically, participants completed a custom five-item questionnaire at the end of each gaming session. This questionnaire was administered directly through the system. A dedicated web interface allowed participants to rate each item on a scale from 1 (low score) to 5 (high score). This rating system corresponded to a 5-point Likert scale. The questions (in Italian) were as follows:

- Q1: How would you rate your overall well-being before the gaming session?
- Q2: How satisfied were you with the gaming session?
- Q3: How much would you like to play these games again?
- Q4: How fatigued do you feel right now?
- Q5: How easy did you find interacting with the system during the gaming session?

Although the standard System Usability Scale (SUS) was not used in this context, because it was considered too long and potentially difficult to understand, the proposed questions were designed to correspond to four key domains of human-computer interaction in clinical settings. Specifically:

- *Acceptability and tolerability*: assessed by the self-perceived general state before the gaming session (Q1) and the perceived physical fatigue afterwards (Q4).
- *Satisfaction*: evaluated through the participant’s overall gratification with the gaming session (Q2).
- *Engagement*: measured by the participant’s self-reported willingness to repeat the exergames (Q3).
- *Usability*: determined by the participant’s perceived ease of interaction and absence of technical difficulties during gameplay (Q5).

### 2.7 Statistical analysis

Statistical analysis was performed using MATLAB (v.2024a, MathWorks, Natick, MA, United States). Descriptive statistics are reported for Motor Functional Parameters (MFP) to characterize the cohort’s performance across sessions and exergames. Symmetry indices and Game-Based Metrics (GBM) are reported only as mean values.

Comparisons between movement planes (frontal vs. lateral) and coordination modes (single-arm, alternated, simultaneous) in the Gym exergame were performed using independent-sample t-tests on aggregated data. The same approach was applied to compare motor and game-based parameters between initial and final levels in the other exergames to quantify performance improvements. Additionally, Pearson’s correlation coefficients (*ρ*) were calculated to determine the relationship between difficulty levels and the achieved game scores. Statistical significance was set at *p* < 0.05.

To evaluate the longitudinal transition across difficulty game levels, a standardized normalization approach was adopted. Since the exergames have different difficulty levels, the Percentage of Completion (C%) was used to balance the contribution of each exergame to the final metric, defined as the ratio of the highest achieved level to the theoretical maximum level of the exergame.

Subsequently, a Normalized Efficiency Index (NEI) was implemented to quantify the rate of progression while accounting for inter-subject variability in session attendance (e.g., participants completed between 5 and 10 sessions). The NEI assesses the temporal progression to reach individual peak performance and was calculated according to Equation (3):


$$\mathrm{NEI}=\frac{{C_{\mathrm{MAX}}}^{2}}{100xS_{\text{TARGET}}}$$ (3)

Where C_MAX_ represents the individual maximum percentage of completion reached during the protocol, and S_TARGET_ is the session number (in range 1–10) in which this maximum was first achieved. The quadratic term was utilized to prioritize full mastery (100% completion) over rapid but partial progression (e.g., reaching 50% quickly but failing to advance further). The NEI metric was computed for each exergame separately and then aggregated to generate a total score (NEI_TOT_) using Equation (4):


$$\mathrm{NE}I_{\mathrm{TOT}}=\mathrm{NE}I_{\text{AIRPLANE}}+\mathrm{NE}I_{\mathrm{SKI}}+\mathrm{NE}I_{\text{PIANO}}+\mathrm{NE}I_{\mathrm{GYM}}$$ (4)

To evaluate the clinical validity of the exergame progressions, the relationship between the NEI_TOT_ and the MDS-UPDRS total and sub-scores was assessed using Spearman’s rank correlation coefficient. This non-parametric measure was preferred due to the ordinal nature of the MDS-UPDRS clinical scale and the small sample size, providing a more robust analysis of the monotonic relationship between variables.

## 3 Results

### 3.1 Overall protocol and technical results

One participant attended only a single gaming session over the four-week study period and was consequently excluded from the analysis. The analysis was conducted on the data collected from the remaining 14 participants who completed five or more sessions: 10 participants completed all 10 scheduled sessions (2–3 sessions per week); 2 participants completed 7 sessions (averaging nearly 2 sessions per week); and 2 participants completed 5 sessions (averaging just over 1 session per week). Table 1 summarizes the demographic and baseline clinical characteristics of the final analyzed cohort (*n* = 14). Of the four participants who completed fewer than 10 sessions, none dropped out of the study or experienced technical difficulties or disengagement with the system. The protocol was designed to be flexible, accommodating participants’ logistical needs and travel availability to the association’s facility. While some participants attended fewer than 10 gaming sessions, they all completed the four-week experimental period and were therefore included in the analysis.

To provide a comprehensive overview of the analysis, Table 2 summarizes key metrics of each exergame. Specifically, the table reports the number of failed recordings (trials not correctly saved by the system), valid recordings (trials available for analysis), the task completion rate (trials successfully completed without premature interruption due to fatigue), and the total engagement time (cumulative duration of completed tasks).

**Table 2:** Summary of exergame technical reliability, task completion rates, and cumulative engagement time.

Suite exergames	Technical performance		Participants’ performance	
Name	Failed recordings	Valid recordings	Completed tasks and rate (%)	Total engagement time
AIRPLANE	2	349	314 (89.97%)	13,707 s (~ 3 h 48 m)
SKI	25	194	184 (94.85%)	38,348 s (~ 10 h 39 m)
PIANO	0	279	279 (99.64%)	10,785 s (~ 2 h 59 m)
GYM (frontal)^1^	1	482	482 (100.00%)	15,159 s (~ 4 h 12 m)
GYM (lateral)^1^	0	486	486 (100.00%)	13,814 s (~3 h 50 m)

^1^Counts include single-arm, simultaneous, and alternating exercise modes.

The system demonstrated high technical stability, as shown in Table 2. The highest number of technical failures occurred with the Ski exergame (*n* = 25), likely due to larger file sizes associated with longer task execution times or potential latency during the transfer of large recordings.

The Ski exergame proved the most demanding, requiring high coordination and significant physical effort. This complexity is reflected in two elements: the highest cumulative time (10 h 39 m), despite having the lowest number of recordings; and the task completion rate (94.85%), suggesting that interruptions were likely due to fatigue or prolonged physical effort required to maintain rhythmic leg movements. In contrast, Piano was the least complex exergame, with a nearly perfect completion rate (99.64%) and a shorter overall duration (2 h 59 m), indicating that participants managed the arm-pointing task and limb extension without excessive difficulty. The Airplane exergame showed the highest percentage of incomplete trials (~10%), likely due to the sustained postural control required: the continuous changes in flight trajectory impose a persistent challenge to trunk control, leading to premature termination as fatigue sets in. Finally, the Gym exergame demonstrated excellent feasibility with a 100% completion rate.

### 3.2 Exergame performance analysis

#### 3.2.1 Airplane exergame: postural and motor performance

As shown in Table 3, completed trials were distributed across difficulty levels 0–8, excluding Level 6, which was bypassed by the clinical team, allowing participants to progress directly to the more challenging levels (7 and 8).

**Table 3:** MFP and GBM mean values for AIRPLANE.

Game info		MFP					GBM			
Level	Trials	ARM_ANG_	ELB_ANG_	ARM_SI_	ELB_SI_	T_ANG_	PAUSE	ERROR	POINT	TIME
0	58	81.7 ± 8.2	147.4 ± 4.7	0.062	0.0215	4.5 ± 1.7	0.04	0.00	5.40	59.44
1	58	87.3 ± 12.1	158.2 ± 5.1	0.001	0.007	3.5 ± 2.3	0.09	0.00	5.61	37.24
2	50	84.6 ± 12.8	159.6 ± 5.3	0.025	0.010	3.9 ± 2.4	0.07	0.00	5.13	30.71
3	7	89.2 ± 13.4	153.3 ± 4.0	0.076	0.105	3.9 ± 2.0	0.00	0.00	7.00	65.50
4	39	85.8 ± 11.7	158.8 ± 4.4	0.004	0.001	3.5 ± 2.5	0.35	0.03	6.21	45.68
5	43	92.8 ± 12.0	161.9 ± 4.7	0.010	0.007	3.9 ± 2.4	0.16	0.08	6.55	35.03
7	50	87.7 ± 12.3	158.9 ± 5.4	0.005	0.013	3.3 ± 2.3	0.10	0.13	9.08	53.94
8	44	89.7 ± 14.06	155.3 ± 4.4	0.009	0.003	3.8 ± 3.2	0.00	0.18	9.23	41.50

In terms of MFPs, the analysis focused on arm and elbow kinematics (ARM_ANG_ and ELB_ANG_) alongside trunk stability (T_ANG_). A statistically significant improvement was observed for the arm angle (ARM_ANG_), which increased from 81.7° at Level 0 to 89.7° at Level 8 (*p* < 0.001). Similarly, the elbow angle (ELB_ANG_) showed high values across all difficulty levels. These elements indicate that participants, in general, were able to maintain the required “T-pose” posture despite the increasing complexity of flight paths and the introduction of additional motor and cognitive stimuli. The lower values recorded at baseline (Level 0) suggest a less correct posture (lower, more flexed arms) compatible with more impaired motor profiles. The symmetry indices (SI) for both arms (ARM_SI_) and elbows (ELB_SI_) remained close to zero, indicating excellent bilateral arm extension during the task. Crucially, trunk stability (T_ANG_) remained remarkably stable; although the average angle decreased, this change was not statistically significant (*p* = 0.15). This is a key clinical finding, as it demonstrates the ability to recover correct trunk posture despite the continuous lateral solicitations required to follow the flight path.

The GBMs effectively captured the increasing task difficulty. A strong, significant positive correlation was found between difficulty level and average points achieved (*ρ* = 0.91, *p* < 0.01), with a range of 5.40 to 9.23. This indicates a substantial improvement in targeting precision despite the introduction of new motor and cognitive challenges at higher levels. Interestingly, the time spent per level showed significant variability, peaking again at Levels 3 and 7, which introduced the take-off movement and longer flight paths combined with higher speeds and cognitive stimuli.

#### 3.2.2 Ski exergame: lower-limb training

The results for the Ski exergame, summarized in Table 4, reflect the high physical demand of this task. Trials were distributed across levels 0–11, though the clinical team bypassed certain intermediate levels (e.g., 3, 6, and 9) to allow rapid progression to more challenging stages based on individual clinical performance.

**Table 4:** MFP and GBM mean values for SKI.

Game info		MFP					GBM			
Level	Trials	LEG_ANG_	KNEE_ANG_	LEG_SI_	KNEE_SI_	T_ANG_	PAUSE	ERROR	POINT	TIME
0	61	109.4 ± 5.7	68.9 ± 7.3	0.035	0.070	2.8 ± 1.5	0.00	2.65	7.33	247.30
1	28	102.4 ± 5.6	76.5 ± 7.2	0.046	0.064	2.7 ± 1.7	0.00	2.29	7.96	193.07
2	4	96.5 ± 4.6	82.0 ± 6.4	0.050	0.089	1.8 ± 1.3	0.00	0.00	8.00	87.25
4	12	104.2 ± 5.7	74.7 ± 7.5	0.033	0.064	2.7 ± 1.5	0.00	3.17	7.58	188.67
5	3	97.9 ± 3.7	80.8 ± 3.7	0.07	0.096	1.3 ± 0.9	0.00	0.00	10.00	63.33
7	16	104.3 ± 5.3	76.1 ± 7.0	0.045	0.072	3.6 ± 2.3	0.00	3.13	8.19	173.31
8	20	109.6 ± 5.1	69.4 ± 7.0	0.047	0.071	2.6 ± 1.9	0.00	2.65	9.00	164.60
10	12	102.6 ± 7.0	78.7 ± 8.2	0.041	0.080	2.9 ± 1.7	0.08	4.83	8.58	275.83
11	38	101.0 ± 5.7	77.9 ± 7.2	0.068	0.083	2.3 ± 1.5	0.00	2.41	9.43	213.62

Regarding MFPs, the parameters focused on lower-limb kinematics. Leg and knee angles (LEG_ANG_ and KNEE_ANG_) showed a dynamic relationship with difficulty: while values remained relatively stable around 101°-109° for the legs and 69°–78° for the knees, slight fluctuations were observed at higher speeds (e.g., Levels 2 and 5), denoting increased difficulty in controlling leg movements during rapid skier progression. Interestingly, the symmetry indices (LEG_SI_ and KNEE_SI_) remained consistently low, though they showed slight fluctuations at higher levels (e.g., 0.096 at Level 5) as fatigue set in. However, these changes were not statistically significant (*p* > 0.05), suggesting that participants maintained a stable bilateral coordination across the most demanding levels of the protocol. Trunk stability (T_ANG_) remained consistently below 4°, indicating that participants performed intensive lower-limb movements from a sitting position without compromising upper-body posture.

GBMs provide further insight into the high engagement required. The time spent per trial remained consistently high, confirming that Ski provided the longest continuous physical stimulation. However, the difference between the initial straight track (Level 0: 247.3 s) and the final circular track (Level 11: 275.83 s) was primarily due to path length rather than decreased motor speed. Despite this challenge, the average number of errors remained relatively stable throughout progression (2.29 to 4.83), even as speed and track length increased, suggesting a slow but effective level of skier control from the very first levels. Furthermore, average points increased significantly from 7.33 to 9.43 at Level 11 (*p* < 0.01). Statistical analysis also revealed a positive correlation between difficulty level and scoring performance (*ρ* = 0.63, *p* = 0.06), suggesting a trend toward greater precision as complexity increased, despite the onset of fatigue.

#### 3.2.3 Piano exergame: upper-limb targeting

The results for the Piano exergame, summarized in Table 5, confirm its role as the least complex task in the protocol, characterized by high efficiency and rapid execution. The trials were distributed across three levels (0–2), showing a progressive improvement in motor performance.

**Table 5:** MFP and GBM mean values for PIANO.

Game info		MFP		GBM			
Level	Trials	ARM_ANG_	T_ANG_	PAUSE	ERROR	KEY_TIME_	TIME
0	87	71.4 ± 15.1	4.8 ± 2.0	0.06	4.08	6.42	32.11
1	79	80.1 ± 16.0	5.1 ± 3.3	0.35	3.00	5.32	37.22
2	112	83.6 ± 16.8	4.7 ± 2.7	0.29	1.88	4.51	45.09

In terms of MFPs, the analysis focused on the arm elevation angle (ARM_ANG_) and trunk stability (T_ANG_). A clear and significant upward trend was observed for ARM_ANG_, which increased from 71.4° at Level 0 to 83.6° at Level 2 (*p* < 0.001), indicating that participants progressively enhanced limb extension. Trunk stability (T_ANG_) remained remarkably consistent, suggesting that the lateral arm movements required to reach target keys did not induce significant postural changes, even during longer key sequences and prolonged execution time of the highest levels.

The GBMs highlight the participants’ increased rapidity and efficiency. KEY_TIME_ (the time elapsed between consecutive key presses) showed a significant progressive reduction (*p* < 0.01), demonstrating faster execution despite increasing sequence complexity. The slight increase in total time is attributable solely to the greater number of keys required at advanced levels. Regarding efficiency, the average number of errors per trial decreased progressively (*p <* 0.05), showing a strong negative correlation with difficulty levels (*ρ* = −0.97, *p* < 0.001). This provides statistical evidence that participants gained more effective control over their arm movements. Finally, the average number of pauses remained low, with only a slight increase noted as participants managed prolonged arm extension during more intense targeting sequences.

#### 3.2.4 Gym exergame: frontal and lateral upper-limb training

The results for the Gym exergame, categorized by movement plane (frontal vs. lateral) and coordination mode (Single, Alternated, Simultaneous), are presented in Tables 6, 7 and allow for a direct comparison of motor performance across different physical demands.

**Table 6:** MFP and GBM mean values for GYM (frontal direction).

Game info			MFP					GBM		
Mode/Level		Trials	ROM_ANG_	ARM_VEL_	ANG_SI_	VEL_SI_	PPM	ERROR	MOV_OK_	TIME
SING	0	34	102.3 ± 6.3	70.2 ± 21.9	0.19	0.34	12.2 ± 5.7	0.68	82.9%	23.24
	1	62	98.7 ± 7.7	91.9 ± 19.5	0.14	0.16	20.5 ± 5.1	0.53	88.3%	27.21
	2	50	97.9 ± 8.9	102.9 ± 23.9	0.15	0.23	24.2 ± 8.1	0.72	89.6%	36.12
	3	94	98.6 ± 10.2	130.9 ± 28.1	0.13	0.20	30.7 ± 8.2	1.30	89.9%	37.87
ALT	0	18	109.6 ± 5.1	87.8 ± 14.5	0.16	0.23	16.9 ± 3.5	0.28	82.2%	16.11
	1	31	105.3 ± 8.4	104.0 ± 26.0	0.13	0.32	18.7 ± 4.4	0.81	98.1%	25.16
	2	24	104.5 ± 8.7	117.0 ± 33.7	0.11	0.35	22.1 ± 6.8	0.67	98.1%	29.79
	3	48	114.2 ± 9.7	154.5 ± 52.8	0.12	0.21	25.8 ± 8.2	2.10	100.0%	33.31
SIM	0	15	108.1 ± 4.8	98.9 ± 20.2	0.06	0.10	23.3 ± 6.0	1.20	93.3%	18.60
	1	34	102.1 ± 7.7	110.8 ± 25.6	0.05	0.16	26.8 ± 6.2	0.62	92.1%	27.59
	2	24	97.0 ± 10.7	126.5 ± 39.3	0.06	0.15	30.9 ± 10.5	1.54	95.6%	36.29
	3	48	103.8 ± 10.2	168.4 ± 59.0	0.06	0.11	36.7 ± 10.9	0.94	90.7%	38.40

SING, Single-arm mode; ALT, Alternated-arm mode; SIM, Simultaneous-arm mode.

**Table 7:** MFP and GBM mean values for GYM (lateral direction).

Game info			MFP					GBM		
Mode/Level		Trials	ROM_ANG_	ARM_VEL_	ANG_SI_	VEL_SI_	PPM	ERROR	MOV_OK_	TIME
SING	0	36	89.0 ± 6.1	72.1 ± 13.9	0.17	0.24	14.6 ± 4.9	0.92	80.0%	17.83
	1	62	76.6 ± 5.7	82.4 ± 13.0	0.12	0.15	23.9 ± 7.2	0.26	87.6%	24.34
	2	50	76.2 ± 6.2	87.4 ± 14.4	0.10	0.19	25.7 ± 11.1	0.46	87.0%	35.30
	3	94	81.4 ± 5.1	120.9 ± 15.0	0.10	0.13	37.0 ± 11.3	0.01	94.3%	33.88
ALT	0	14	87.1 ± 7.1	77.8 ± 10.7	0.11	0.21	17.5 ± 4.3	0.86	100.0%	17.35
	1	34	82.1 ± 7.3	87.6 ± 14.7	0.10	0.17	19.3 ± 5.1	0.56	99.4%	24.00
	2	25	76.7 ± 5.7	89.0 ± 14.5	0.12	0.17	21.0 ± 5.2	0.04	97.1%	28.64
	3	48	86.1 ± 5.9	115.6 ± 19.8	0.10	0.17	26.7 ± 7.6	0.10	97.0%	29.35
SIM	0	18	86.6 ± 5.3	75.9 ± 14.7	0.08	0.25	24.5 ± 4.8	1.00	89.4%	11.50
	1	31	77.3 ± 6.4	90.0 ± 15.9	0.08	0.08	30.7 ± 7.7	0.45	90.0%	22.87
	2	25	73.0 ± 6.1	88.2 ± 16.7	0.12	0.23	31.2 ± 10.2	0.20	93.4%	34.08
	3	48	80.6 ± 5.1	128.2 ± 22.5	0.09	0.11	42.4 ± 13.8	0.02	96.1%	34.75

SING, Single-arm mode; ALT, Alternated-arm mode; SIM, Simultaneous-arm mode.

In terms of MFPs, participants achieved significantly greater Range of Motion (ROM_ANG_) during frontal exercises than during lateral movements (*p* < 0.001). This difference suggests that frontal movements were more naturally executed. A similar trend was observed for movement speed (ARM_VEL_): the frontal mode elicited significantly faster movements (*p* < 0.001), whereas lateral movements remained slower and more constrained. This behavior can be attributed to the direct visual feedback available during frontal execution, which allows participants to easily monitor their arm position. In contrast, lateral movements are performed “blindly,” requiring greater reliance on proprioception. Notably, the Symmetry Indices (ANG_SI_ and VEL_SI_) were consistently lower in the simultaneous mode across both directions, indicating that bimanual synchronized tasks promote better motor symmetry than single-arm or alternated tasks in Parkinson’s patients.

The GBMs highlight an interesting trade-off between speed and accuracy. While the frontal direction allowed higher speeds, it also led to a higher average number of errors and a slightly lower percentage of correct movements (MOV_OK_), particularly at higher difficulty levels. In contrast, the lateral direction, despite reduced ROM and speed, demonstrated superior precision, with MOV_OK_ rates frequently exceeding 95% and average errors approaching zero at advanced levels.

The PPM parameter (Peaks Per Minute) confirms a high level of engagement and efficiency, especially in the lateral direction, where PPM is consistently higher (*p* < 0.001). Additionally, the Simultaneous-arm mode (SIM) showed the highest PPMs, suggesting that synchronized bimanual activity is not only more symmetrical but also more productive. These results suggest that the two movement directions serve complementary rehabilitative purposes: while frontal movements are more effective for training mechanical power and maximum range, lateral movements are ideal for refining coordination, rhythm, and executive precision.

### 3.3 Participants’ progression through difficulty levels

The participants’ progression through the difficulty levels of the four exergames is summarized in Table 8, which reports the maximum level achieved by each subject and the specific session in which that goal was first reached. These data provide a direct measure of the participants’ ability to adapt to the increasing motor and cognitive demands of the protocol.

**Table 8:** Maximum difficulty levels reached by each participant.

Subject	AIRPLANE Max level (Session)	SKI Max level (Session)	PIANO Max level (Session)	GYM Max level (Session)
S01	8 (5)	11 (5)	2 (4)	3 (4)
S02	7 (9)	11 (10)	2 (4)	3 (9)
S05	7 (9)	10 (9)	2 (7)	3 (7)
S06	8 (10)	10 (9)	1 (9)	3 (7)
S07	0 (1)	11 (6)	2 (5)	2 (2)
S10	7 (9)	11 (9)	2 (5)	3 (5)
S13	8 (8)	11 (5)	2 (3)	3 (5)
S14	8 (4)	11 (4)	2 (2)	3 (3)
S17	0 (1)	1 (9)	1 (8)	2 (7)
S18	8 (7)	11 (5)	2 (2)	3 (4)
S19	3 (5)	0 (1)	2 (5)	1 (2)
S20	8 (10)	11 (10)	2 (5)	3 (6)
S21	8 (9)	11 (9)	1 (2)	3 (6)
S22	8 (7)	11 (6)	2 (4)	3 (5)

Overall, the cohort demonstrated strong progression, with most participants reaching the highest difficulty levels well before the end of the 10-session program. In the Ski and Airplane exergames, the most physically and cognitively demanding tasks, participants achieved average maximum levels of 9.4 ± 3.7 and 6.1 ± 3.1, respectively. Notably, for Ski, 78% of participants reached the highest difficulty (Level 11), as early as the 4th or 5th session (e.g., S14 and S18), highlighting rapid adaptation to lower-limb coordination requirements. In the Piano and Gym exergames, which focused on fine targeting and upper-limb range of motion, almost all participants (92%) successfully reached the highest levels (Level 2 for Piano and Level 3 for Gym) within the first half of the training period, typically between sessions 2 and 5. Only a small subset of participants (e.g., S17 and S19) showed a more limited progression in specific games, reflecting the clinical heterogeneity typical of Parkinson’s disease.

**Figure 2:**
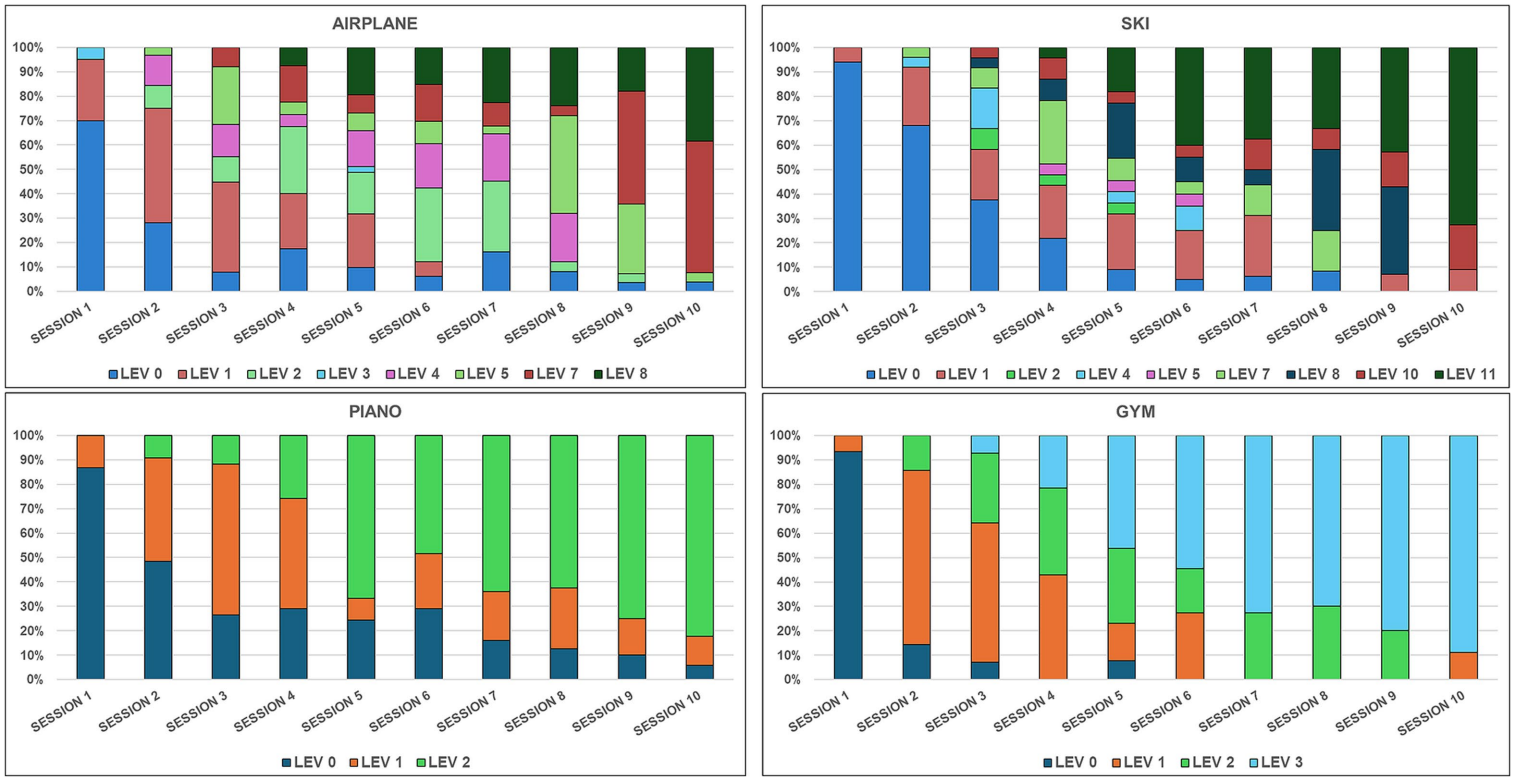
Percentage distribution of trials across difficulty levels for each exergame, to highlight the continuous participants’ progression to more challenging game levels.

Beyond the maximum levels achieved, continuous progression is further evidenced by the percentage distribution of trials completed at each difficulty level across the gaming sessions (Figure 2). The figure highlights a clear shift toward greater difficulty. During the first three sessions, the trials focused on entry-level stages, whereas advanced levels became predominant in the final sessions. Interestingly, across all exergames, a small percentage of trials at lower levels persisted even in the final sessions, attributable to a subset of participants who were unable to progress as rapidly as the rest of the cohort within the 10-session protocol. This finding suggests that while the protocol was effective for most subjects, some may have required a longer training period to achieve a comparable level of progression.

### 3.4 Normalized efficiency index analysis

**Figure 3:**
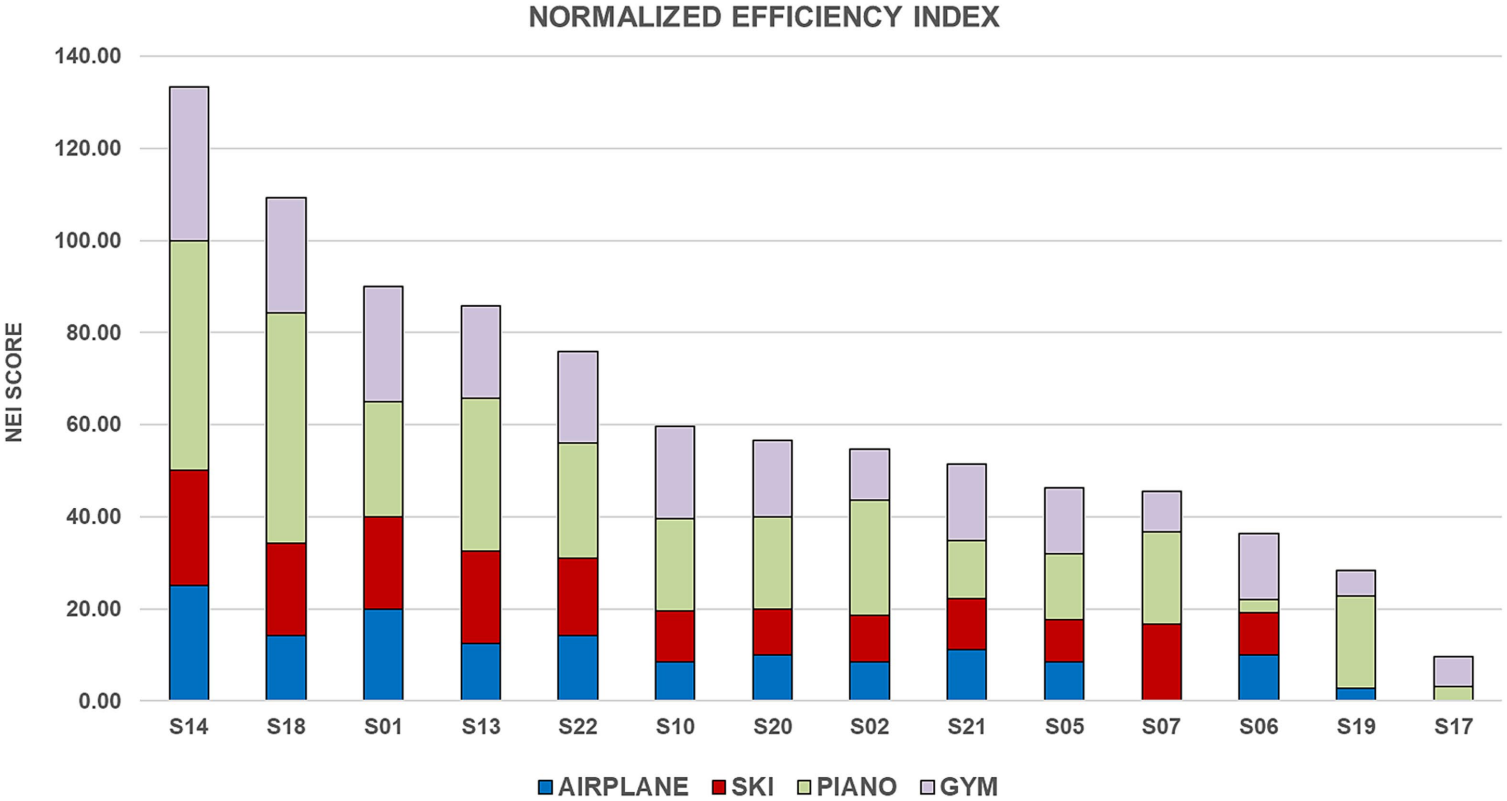
Distribution of the normalized efficiency index (NEI) per subject. Each bar represents the cumulative efficiency score across the four exergames. The segmented colors indicate the relative contribution of each task (Airplane, Ski, Piano, and Gym) to the total performance.

The overall efficiency of the individual progression is summarized by the Normalized Efficiency Index (NEI), as shown in Figure 3. The analysis revealed a broad spectrum of responsiveness across the cohort. S14 and S18 emerged as the top performers, with the highest NEI_TOT_ scores. These “high responders” maintained exceptional efficiency in all exergames. Conversely, the lowest scores were observed for subjects S17 and S19, consistently with the limited progression documented in Table 8 in all exergames, likely reflecting a higher degree of motor impairment.

Interestingly, the contribution of each exergame to the total efficiency varied significantly. While the Piano and Gym exergames provided a consistent baseline of efficiency for nearly all participants, the Airplane and Ski exergames were more discriminative, as higher NEI values were only achieved by a subject subgroup.

These findings confirm that the protocol was accessible, capable of challenging patients at different functional stages, promoting a general positive response, and ensuring that even those with lower overall progression could achieve measurable efficiency.

### 3.5 Correlation between NEI total score and clinical assessment

To assess the clinical validity of the observed progression summarized by the efficiency index, a correlation analysis was performed between NEI_TOT_ and the initial UPDRS clinical assessments. The analysis showed that all correlation values were negative, consistent with the fact that greater game progression, as reflected in higher NEI scores, is inversely related to UPDRS scores, where higher values indicate greater motor impairment.

The most notable result concerned limb mobility (MOBILITY_SCORE_), with a moderate negative correlation (*ρ* = −0.49, *p* = 0.07). This suggests that NEI_TOT_ is particularly sensitive to capturing motor difficulties in the upper and lower limbs, which are the primary body segments engaged by the exergame suite. Furthermore, a general negative trend was observed with the UPDRSIII_SCORE_ (*ρ* = −0.34, *p* > 0.05). Although the coefficient did not reach statistical significance, its direction indicates that participants with lower motor impairment tended to achieve superior progression efficiency. Conversely, no significant correlations were found with other sub-scores (GAITPOS_SCORE_, BRADY_SCORE_, TREMOR_SCORE_, and RIGIDITY_SCORE_).

A deeper analysis of the clinical profiles revealed that subject S18 exhibited a performance profile in sharp contrast to the initial MDS-UPDRS assessment. The subject showed progression comparable to the highest-performing profiles despite a clinical evaluation indicating severe motor impairment (NEI_TOT_ = 109.29, UPDRSIII_SCORE_ = 41). Further investigation revealed that the subject’s clinical assessment was conducted during a wearing-off phase (~210 min post-dose), whereas the gaming sessions occurred during full medication efficacy (~60 min post-dose).

Given that this outlier significantly influenced the overall correlation, the analysis was re-executed, excluding subject S18. This refinement substantially improved the results: a strong, statistically significant correlation emerged between the NEI_TOT_ and both UPDRSIII_SCORE_ (*ρ* = −0.61, *p* = 0.027) and MOBILITY_SCORE_ (*ρ* = −0.66, *p* = 0.014). Additionally, correlations improved for GAITPOS_SCORE_ (*ρ* = −0.36, *p* = 0.228), BRADY_SCORE_ (*ρ* = −0.37, *p* = 0.211), and RIGIDITY_SCORE_ (*ρ* = −0.33, *p* = 0.276): while these correlations did not reach full significance, they demonstrate the importance of considering also these sub-scores in future research. The only correlation that remained unchanged was for TREMOR_SCORE_ (*ρ* = 0.08, *p* = 0.793), which remained negligible. This indicates that tremors, primarily at rest, do not interfere with the functional execution of the exergames.

### 3.6 Feasibility, usability, and engagement outcomes

Overall, the analysis of the session-by-session questionnaires revealed highly positive feedback from all 14 participants, confirming the feasibility and acceptance of the proposed exergaming system. Analyzing the results according to the four predefined domains revealed the following outcomes:

- *Acceptability and tolerability*: Regarding Q1, participants generally reported a good baseline state of well-being state before the gaming sessions (mean score: 3.86 ± 0.80). Importantly, perceived physical fatigue after the gaming sessions (Q4) remained low to moderate (mean score: 2.89 ± 1.28), demonstrating that the physical effort required by the exergames was well tolerated and appropriately tailored to the PD cohort.
- *Satisfaction*: Overall satisfaction with the gaming sessions (Q2) was consistently high, with patients giving an average score of 4.22 ± 0.71.
- *Engagement*: Reflecting a strong motivational appeal (Q3), participants expressed a high willingness to repeat the exergaming experience (mean score: 4.53 ± 0.68).
- *Usability*: Interacting with the system and game interface was intuitive (Q5). Participants reported high ease of use when interacting with the system during the gaming sessions (mean score: 3.52 ± 0.98), indicating the system’s high usability and overall accessibility.

**Figure 4:**
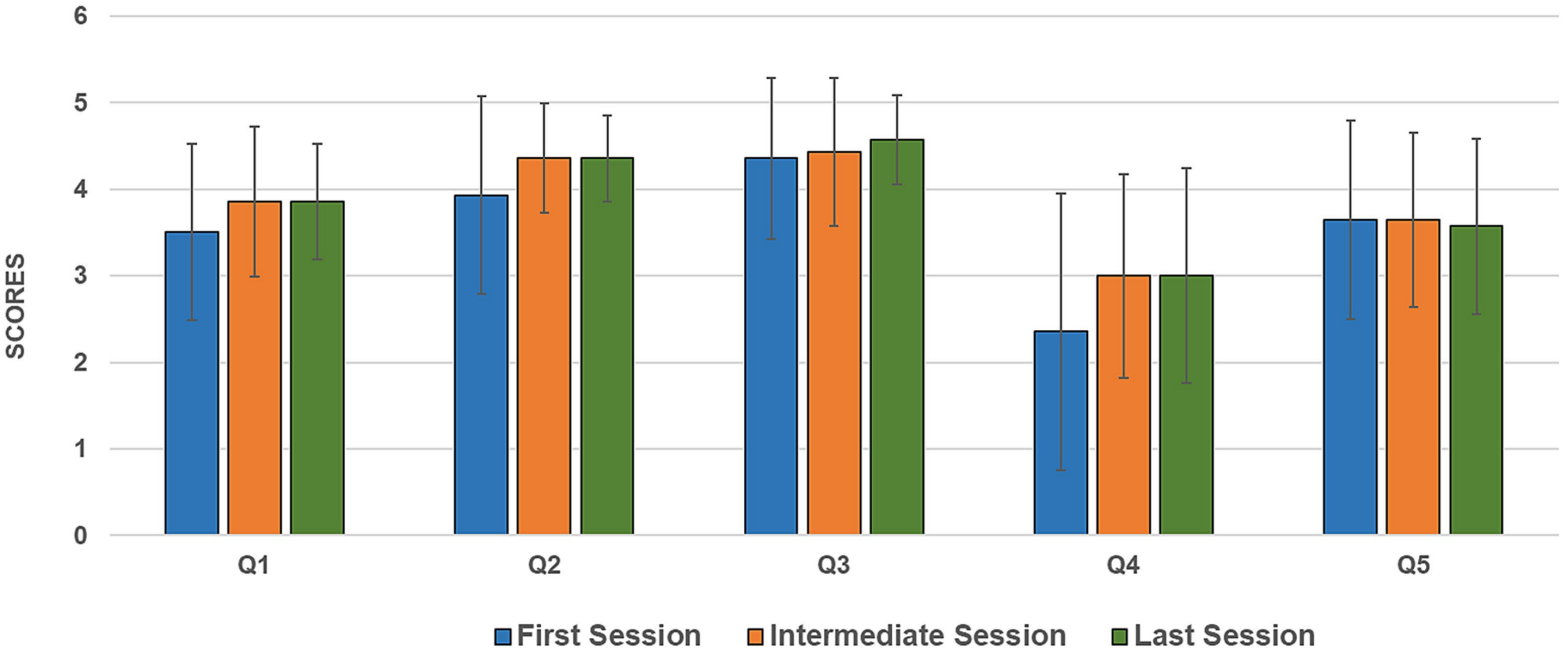
Temporal evolution of the five administered items (Q1–Q5) across the exergaming protocol. Each bar represents the mean scores (with standard deviation) assigned by participants at three specific time points: initial, intermediate, and final sessions.

Analyzing the temporal evolution of the item scores across the exergaming protocol (initial, intermediate, and final sessions) revealed specific trends and relevant insights (Figure 4). Perceived well-being before the gaming session (Q1) increased progressively as the protocol advanced. The same positive trend was observed for overall satisfaction (Q2) and engagement (Q3). Usability (Q5) remained relatively constant, showing only a slight decrease in the final session, likely due to the more challenging in-game levels that participants faced in the latter gaming sessions. Similarly, perceived physical fatigue (Q4) increased from the intermediate session onward. This physiological response is entirely consistent with the increasing of the game’s difficulty and the greater physical demands participants faced from the intermediate session onward.

Mean scores assigned by participants across the gaming sessions reveal further insights and allow us to identify which participants appreciated the system and which did not. Specifically, S14 gave the highest score in all sessions for perceived general well-being (Q1), satisfaction (Q2), and engagement (Q3), as well as a high score (mean score: 4.7) for usability (Q5). Furthermore, this subject assigned a very low score to Q4 (mean score: 1.6), indicating low perceived physical fatigue at the end of the gaming sessions. These ratings align with the optimal progression in exergames of S14, as indicated by the NEI index. In contrast, S17 was the most critical participant, with mean scores of 3.5 for satisfaction (Q2) and 3.9 for engagement (Q3). This subject reported one of the highest levels of perceived fatigue (mean score: 4.2), and the lowest scores for usability (mean score: 2.5) and perceived well-being (mean score: 3.1). This outcome is consistent with the poorest motor profile indicated by the NEI index for S17. Subject S20 was found to be the most fatigued (Q4), with a mean score of 4.8. Despite this, engagement (Q3) was very high (mean score: 4.8), suggesting that the participant was highly motivated to play. Another peculiar case is subject S18, who emerged from the NEI analysis as an anomalous case. This subject showed one of the lowest self-evaluations of perceived well-being (mean score: 3.9) and fatigue (mean score: 1.9), demonstrating once again a discrepancy between the perception of well-being and actual motor performance.

The findings from the analysis of individual participant responses appear to align with the game progression profiles represented by the NEI index. This suggests that responses to questionnaire items could be implicitly influenced by perceived performance during the gaming session. To obtain objective evidence for this hypothesis, we analyzed the correlation between the mean item scores assigned by participants and their NEI progression index using Spearman’s coefficients. The results demonstrate moderate positive correlations between game progression and pre-session general state (*ρ* = 0.45), satisfaction (*ρ* = 0.55), and usability (*ρ* = 0.40). Conversely, there is a moderate negative correlation with post-session fatigue (*ρ* = −0.49), confirming that higher perceived fatigue is associated with lower progression. Engagement (Q3), however, shows only a weak-to-moderate correlation with game progression (*ρ* = 0.39), indicating that patient engagement is relatively independent of actual performance.

Taken together, these self-reported outcomes suggest that the system is not only safe and technically accessible, but also highly capable of maintaining patient motivation and engagement over a multi-week training period.

## 4 Discussion

The training system presented in this study was designed as an integrated exergame platform to bridge the gap between traditional physical therapy and home-based digital health, promoting continuous, personalized training. Although the system was designed for effective and safe use in unsupervised home environments, this preliminary study was conducted at a local patient association facility to preliminarily assess feasibility, usability, and patient feedback.

Exergaming in Parkinson’s disease (PD) offers a unique advantage by combining physical exercise with high-level engagement and cognitive demands (dual tasking). By utilizing four distinct games (Airplane, Ski, Piano, and Gym), the proposed solution targets a wide range of motor domains, including postural control, bilateral coordination of upper and lower limbs, and pointing precision. The technical backbone of this system is the integration of Google MediaPipe Pose (GMP) markerless framework, which represents a significant step forward in remote monitoring. Unlike traditional solutions that require complex cameras (RGB-D devices) or wearable sensors (which can be cumbersome for PD patients), GMP allows for motion body tracking using a simple RGB camera. This technology proved capable of capturing subtle kinematic variables (28, 57, 58) with sufficient accuracy for training and rehabilitation purposes.

Our data on level progression (Table 8) and trial distribution (Figure 2) provide evidence that participants can adapt to digital solutions. We observed a rapid transition from basic to advanced levels, often within the first five sessions. This indicates a high level of engagement and rapid motor adherence to more demanding challenges, according to each individual’s skills and general conditions. The rapid transition noted in Piano and Gym suggests that these exergames are excellent for building confidence and baseline coordination. In contrast, the Ski and Airplane exergames served as functional performance discriminators. However, even participants with lower motor profiles can benefit from a protocol that stimulates neuroplastic mechanisms, although they may require longer protocols to achieve comparable progression (59).

The core innovation lies in the Normalized Efficiency Index (NEI), which does not merely count game points but evaluates how effectively a patient navigates the trade-off between movement quality (kinematics) and task success across increasing levels of difficulty. By analyzing the NEI_TOT_, we observed that the performance progression reflects the patient’s motor status. After excluding outliers influenced by medication timing, we found a strong, significant correlation with the MDS-UPDRS Part III initial assessment scores (*ρ* = −0.61, *p* = 0.027). Particularly striking is the correlation with overall limb mobility (MOBILITY_SCORE_) (*ρ* = −0.66, *p* = 0.014), which suggests that the NEI is exceptionally sensitive to the specific motor impairments that impact daily functional life. This confirms that the exergames are not just games but targeted stressors that reveal the patient’s underlying motor capabilities. Interestingly, the lack of correlation with tremor sub-score suggests that this digital metric captures a different dimension of the disease: the NEI focuses on how a patient actually performs a task, offering a complementary functional perspective to the clinical assessment.

The case of subject S18 offers a relevant insight for future digital health applications. This participant was clinically categorized as severely impaired (UPDRS = 41) but performed like a top user in the games. This discrepancy was not an error in our system but a reflection of the typical On–Off pharmacological cycle (fluctuations). While the clinical assessment captured the subject in a wearing-off phase, the exergame sessions were performed at their functional peak. This highlights a transformative advantage of a digital solution: it could provide a continuous, longitudinal daily view of the patient’s best possible performance in a domestic environment, rather than a single snapshot in a hospital setting.

Finally, the results from the Gym exergame highlight the importance of sensory feedback. We found that while frontal movements (within the participant’s visual field) allowed greater power and speed, lateral movements (outside the participant’s visual field) forced patients to rely more on proprioception. This suggests that the system can be used to specifically retrain the somatosensory system, which is often impaired in PD. By removing direct visual confirmation, we encouraged the brain to listen more closely to the body’s internal signals, a crucial element for improving balance and preventing falls.

The session-by-session administration of the questionnaire provided relevant insights into the participants’ perspectives and the overall feasibility of the exergame-based system. High satisfaction (Q2) and engagement (Q3), along with low-to-moderate fatigue (Q4) and high usability (Q5), demonstrate that the exergame-based protocol was both highly motivating and physically well-tolerated by the PD cohort. Correlation analysis of self-reported scores and objective motor progression (NEI index) revealed a significant psychophysical dynamic. Patient satisfaction (Q2) and pre-session well-being (Q1) were positively correlated with motor progression. This suggests a positive feedback loop. Better in-game performance enhances overall gratification and perceived condition. Conversely, the negative correlation between progression and post-session fatigue (Q4) shows that patients who struggled more with game levels experienced greater physical exertion. Interestingly, engagement (Q3) remained high across the cohort. It showed only a weak-to-moderate correlation with motor progression. From a rehabilitative perspective, this is crucial, as it implies that gamified exercises motivate patients regardless their motor capabilities or the physical effort they expend.

Despite the promising results, this study has some limitations that should be acknowledged. First, despite the large volume of collected data (Table 2), the sample size (N = 14) is relatively small and clinically heterogeneous. Additionally, the cohort was limited to individuals with preserved cognition to guarantee the safe use and effective comprehension of the proposed system, without explicitly evaluating cognitive status using standard clinical scales, such as the Montreal Cognitive Assessment (MoCA) or the Mini-Mental State Examination (MMSE). Taken together, these factors suggest that a larger cohort, also exploring the effects of cognitive decline, would be necessary to fully generalize the findings to the broader PD population and to strengthen the statistical power of the correlations with clinical scales. Another limitation of the study is the absence of clinical follow-up assessments at the end of the protocol and of long-term follow-up assessments. Participants were evaluated with the MDS-UPDRS motor examination only at baseline (T0) to characterize their clinical profile. Since this pilot study primarily aimed to evaluate feasibility, usability, and the in-game kinematic progression over a relatively short period (up to 4 weeks), it was not designed to capture clinical changes on standard scales. Future randomized controlled trials with longer intervention periods, larger cohorts, and rigorous fixed-session protocols and follow-up milestones are needed to determine whether the observed in-game motor improvements translate into clinically meaningful benefits and improvements in daily life. Another limitation concerns the participants’ pharmacological state. As demonstrated by subject S18, the timing of medication intake can significantly affect performance and clinical scores, potential biasing in the correlation analysis. In contrast, the system was able to automatically detect potential incongruence that could become fundamental for daily fluctuation monitoring. Additionally, an intrinsic limitation of the proposed markerless exergaming system, and of home-based telerehabilitation solutions in general, is the inherent lack of tactile feedback and physical guidance. Although these systems typically provide real-time visual and auditory cues to guide patients in performing correct movements, they cannot offer manual corrections of body position and posture, which are provided by physical therapists during conventional in-person rehabilitation. This hands-on guidance is often crucial for people with Parkinson’s disease, especially when complex postural abnormalities are present, to ensure optimal movement execution. In a home setting, although the therapist is not physically present, patients with more evident postural alterations can still be supported by a caregiver who can verbally prompt or physically demonstrate the correct posture and movements. From this perspective, such technological interventions should be viewed as complementary tools that enhance the continuity of care, rather than as replacements for conventional, therapist-guided, in-person sessions. Finally, the current analysis primarily focuses on voluntary motor control and game achievements, without explicitly accounting for involuntary complications such as tremor or dyskinesia, which are common in PD. Future developments of the proposed solution could integrate high-frequency signal analysis from GMP to isolate and quantify other aspects of the disease, providing an even more comprehensive digital footprint of the individual condition.

## 5 Conclusion

This study demonstrates that a customized suite of exergames, integrated with markerless motion tracking (Google MediaPipe Pose), provides a reliable and engaging platform for Parkinson’s disease functional training. The introduction of the Normalized Efficiency Index represents a significant metric to objectively characterize individual progression, as it successfully correlates digital performance with standardized clinical scales (UPDRS). Our results show that patients can successfully progress to higher difficulty levels depending on their individual motor condition, with high satisfaction and engagement. Furthermore, the system’s ability to capture functional performance offers a comprehensive view of the patient’s potential. Future research with larger cohorts and longitudinal follow-ups will further define the role of exergames in personalized functional training protocols.

## Data Availability

The datasets presented in this study can be found in online repositories. The names of the repository/repositories and accession number(s) can be found at: doi: 10.5281/zenodo.18400034, Zenodo.
