# Supplementary material for: Digital monitoring of motor function in Parkinson’s disease using Markerless motion analysis and exergaming
Source: Front Neurol. 2026 Apr 15;17:1800332. doi: 10.3389/fneur.2026.1800332 (PMC13130081; doi:10.3389/fneur.2026.1800332)
Supplement: Supplementary file 1 [file Supplementary_File_1.docx]

Supplementary Material

# AIRPLANE Exergame

In the AIRPLANE exergame, the participant guides a plane avatar using trunk movements, following a flight path marked by circles while avoiding mountain obstacles. Performed in a seated position, the exercise requires the user to keep their arms laterally extended as much as possible. Lateral trunk movements are used to execute the turns necessary to pass through the target circles that define the flight trajectory. Supplementary Figure 1 illustrates the main scenario, the avatar, and the required posture for controlling the aircraft. If the participant lowers the arms during execution (e.g., due to excessive fatigue), the game automatically enters a Pause mode. Gameplay resumes from the same point once the correct posture is restored.

**
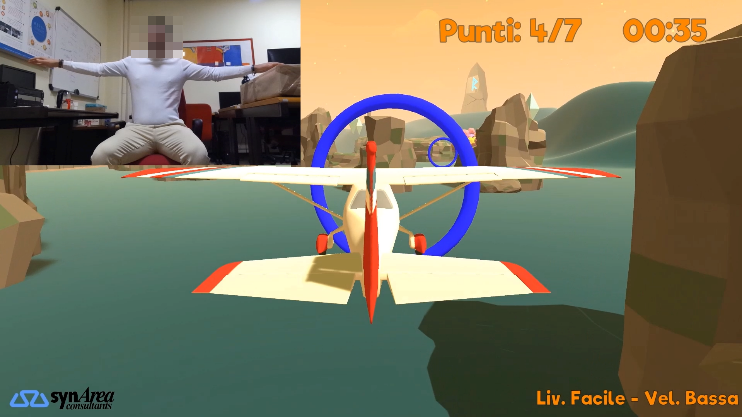
**

**Supplementary Figure 1**. Main scenario for the AIRPLANE exergame: the correct posture is shown in the top-left corner.

The primary objective is to complete the flight path while accumulating the highest score by passing through the target circles. Colliding with an obstacle ends the trial. At those levels where the cognitive stimulus is active, distractors are introduced as additional circles in different colors; passing through a wrong-colored circle counts as an error.

The exergame is structured across 9 difficulty levels (Table S1), which vary based on game configuration parameters (flight dynamics and flight path) and the physical movement required:

- Levels 0–2: The aircraft starts already in flight (no take-off required). The trajectory is defined by 7 target circles, and the cognitive stimulus is not active. The difference between the three levels is the flight speed (Low, Medium, High), controlled by an internal factor that modifies the aircraft's dynamics (pitch, roll, linear velocity) and sensitivity as defined by the Unity physics engine.
- Levels 3–5: These levels introduce the take-off, executed via a backward-forward trunk movement in the initial phase. The flight path is extended to 8 target circles, and the cognitive stimulus is active (5 distractors). Flight speed varies across levels (Low, Medium, High), as previously described.
- Levels 6–8: Both take-off and cognitive stimuli are yet present. The flight path is extended, featuring 11 target circles and an increased number of distractors (6). Speed varies across levels (Low, Medium, High), as previously described.

**Table S1.** Game levels for AIRPLANE Exergame

| **Game Level** | **Speed** | **Target circles** | **Take Off** | **Cognitive Stimulus (Distractor circles)** |
| --- | --- | --- | --- | --- |
| 0 | Low | 7 | No | No |
| 1 | Medium | 7 | No | No |
| 2 | High | 7 | No | No |
| 3 | Low | 8 | Yes | Yes (5) |
| 4 | Medium | 8 | Yes | Yes (5) |
| 5 | High | 8 | Yes | Yes (5) |
| 6 | Low | 11 | Yes | Yes (6) |
| 7 | Medium | 11 | Yes | Yes (6) |
| 8 | High | 11 | Yes | Yes (6) |

# SKI Exergame

In the SKI exergame, the participant controls a skier avatar through alternating leg movements while following a snowy path delimited by wooden fences. Performed in a seated position, the exercise requires the coordination of leg movements to maintain correct alternation and a straight trajectory. Arm raises are used to control and change the skier's direction. To provide incentives and objectives, gems are distributed along the track. Supplementary Figure 2 illustrates the main scenario, the avatar, and the required posture for controlling the skier. If the participant lowers the arms during execution (e.g., due to excessive fatigue), the game automatically enters a Pause mode. Gameplay resumes from the same point once the correct posture is restored.

**
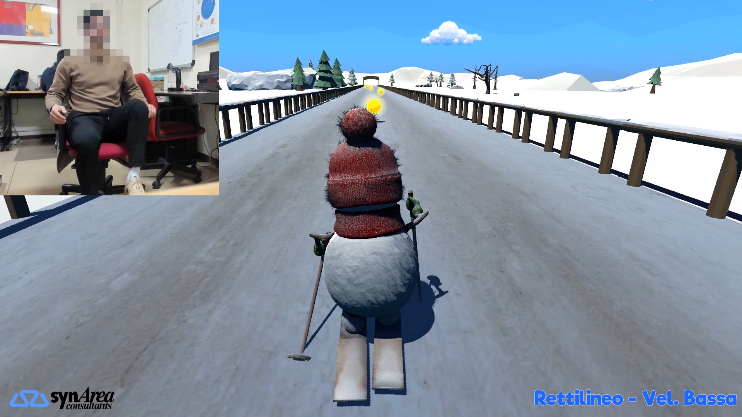
**

**Supplementary Figure 2**. Main scenario for the SKI exergame: the skier avatar and the snowy track environment. The reference posture for the exercise is shown in the top-left corner.

The primary objective is to complete the track while accumulating the highest score by collecting gems. Colliding with the lateral wooden fences is recorded as an error. No explicit cognitive stimuli (e.g., distractors) have been included in this specific exergame, focusing instead on physical coordination.

The exergame is structured across 12 difficulty levels (Table S2), which vary based on game configuration parameters (skier dynamics and track complexity) and the physical movement required. The number of gems remains fixed across all levels to maintain a consistent scoring baseline.

- Levels 0–2: The track is straight, requiring no changes in direction (no arm movements). The difference between these levels is the skier’s speed (Low, Medium, High), controlled by an internal factor that modifies the avatar's dynamics and sensitivity, as defined by the Unity physics engine.
- Levels 3–5: These levels introduce a Curved track, consisting of an initial straight path followed by a right-hand curve. Consequently, an explicit change of direction is required by raising the right arm. Additional arm raises may be necessary to adjust the skier’s trajectory after the curve. Skier speed varies across levels (Low, Medium, High).
- Levels 6–8: These levels feature an S-shaped track, with straight sections interspersed with a right turn followed by a left turn. Explicit changes of direction require raising the right and left arms, respectively. Further arm raises may be required for trajectory correction. This track is overall longer than the previous types, requiring greater and more prolonged physical effort. Skier speed varies across levels (Low, Medium, High).
- Levels 9–11: These levels introduce a Closed track, consisting of a ring path with multiple right-hand curves. Explicit changes of direction are necessary by repeatedly raising the right arm. Further arm raises may be required for trajectory correction. This track is the longest in the set and demands the highest level of physical endurance. Skier speed varies across levels (Low, Medium, High).

**Table S2.** Game levels for SKI Exergame

| **Game Level** | **Speed** | **Number of gems** | **Track** |
| --- | --- | --- | --- |
| 0 | Low | 10 | Straight |
| 1 | Medium | 10 | Straight |
| 2 | High | 10 | Straight |
| 3 | Low | 10 | Curved |
| 4 | Medium | 10 | Curved |
| 5 | High | 10 | Curved |
| 6 | Low | 10 | S-shape |
| 7 | Medium | 10 | S-shape |
| 8 | High | 10 | S-shape |
| 9 | Low | 10 | Closed |
| 10 | Medium | 10 | Closed |
| 11 | High | 10 | Closed |

# PIANO Exergame

In the PIANO exergame, the participant controls a virtual-hand avatar by moving the arm through pointing, lateral, and vertical movements. Performed in a seated position, the exercise requires high precision to direct the virtual-hand trajectory toward the target key on the keyboard, followed by a down-up arm movement to press it. Once the key is pressed, the participant moves the arm toward the next key in the sequence. Supplementary Figure 3 illustrates the main scenario, the virtual-hand avatar, and the required posture. If the participant lowers the arm (e.g., due to excessive fatigue), the game automatically enters a Pause mode. Gameplay resumes from the same point once the correct posture is restored.

**
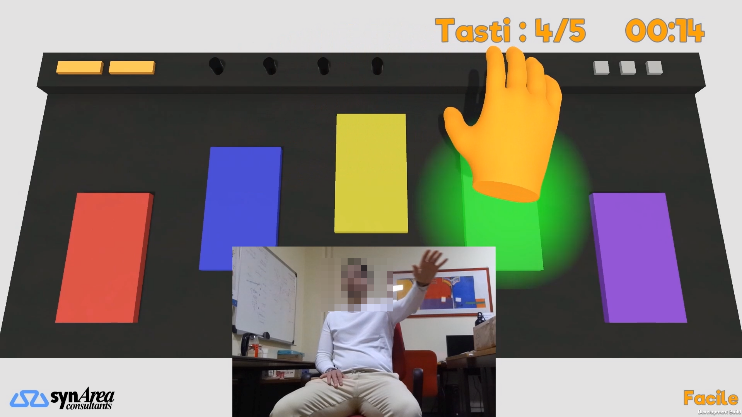
**

**Supplementary Figure 3**. Main scenario for the PIANO exergame: the virtual-hand avatar and the piano interface. An example of an illuminated target key is shown, which the participant must press while avoiding inactive keys.

The primary objective is to complete the sequence of keys while minimizing errors caused by pressing inactive keys, whether voluntary or involuntary. Pressing an incorrect key results in an error. Such errors may arise from two factors: a cognitive failure to correctly identify the target key or a motor failure to properly lift the arm, resulting in an unwanted sliding effect across multiple keys. Although no explicit distractors are present, this exergame imposes an implicit cognitive demand on motor planning and spatial mapping.

The exergame is structured across 3 difficulty levels (Table S3), which differ solely by the length of the key sequence (5, 7, or 10 keys). The core challenge of this exergame lies in the precision of movement and the avoidance of errors: since the task requires substantial and prolonged arm postural control, the game levels were limited.

**Table S3. Game levels for PIANO Exergame**

| **Game Level** | **Length of the key sequence** |
| --- | --- |
| 0 | 5 |
| 1 | 7 |
| 2 | 10 |

# GYM Exergame

In the GYM exergame, the participant controls the upper limbs of a "sticky-human" avatar. Depending on the user's postural stability, the exercise can be performed in either a seated or standing position. The exergame requires the participant to perform accurate, synchronized arm raises across three distinct modalities: repetitive single-arm movements (right and left separately), alternating arm movements, and simultaneous bilateral movements. The game features two movement directions: frontal raises and lateral raises. This distinction was implemented because the direction of movement often represents an additional challenge for patients with specific motor pathologies. Supplementary Figure 4 illustrates the main scenario, the avatar, and the required posture. The eight movement sequences (4 for frontal and 4 for lateral directions) are proposed consecutively; however, the session can be interrupted at any time due to excessive fatigue.

**
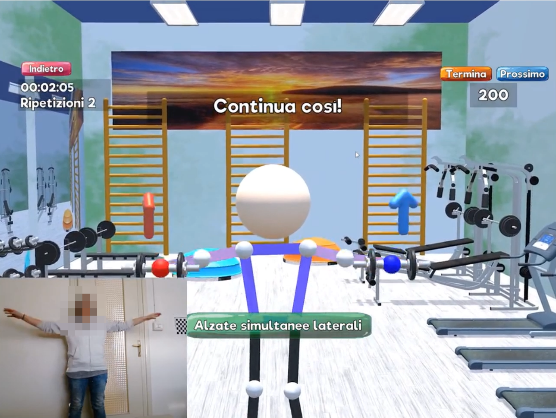
**

**Supplementary Figure 4**. Main scenario for the GYM exergame: the sticky-human avatar within the gym environment. The figure demonstrates an example of simultaneous lateral arm raises.

The primary objective is to complete the sequence of movements and achieve the highest by counting the number of correct movements in the designated direction. Movements performed in the wrong direction or with incorrect coordination are recorded as errors. Currently, while a parametrization of the minimal raising angle has not yet been included, the game uses a fixed 60-degree threshold (the angle between the arm and the trunk) to evaluate a movement as "sufficient" or "poor." Although no explicit distractors are present, this exergame involves a significant implicit cognitive demand, particularly during bilateral executions, as the participant must adhere to the specific coordination pattern required.

The exergame is structured across 4 difficulty levels (Table S4), which differ solely by the length of the movement sequence (5, 10, 15, or 20 repetitions). Due to the prolonged physical effort required for upper limb elevation and speed, the number of levels was intentionally limited. Additionally, a time constraint is implemented: a maximum of 4 seconds per movement is configured to ensure that repetitions are performed within an established time window.

**Table S4.** Game levels for GYM Exergame

| **Game Level** | **Number of repetitions** |
| --- | --- |
| 0 | 5 |
| 1 | 10 |
| 2 | 15 |
| 3 | 20 |

# List of motor functional parameters (MFP) and game-based performance metrics (GBM)

This section shows the list of all MFPs and GBMs considered for each exergame.

**Table S5.** List of MFPs and GBMs

| **Exergame** | **Parameter** | **Description** | **Computation^1^** |
| --- | --- | --- | --- |
| AIRPLANE | ARM_ANG_ (deg) | Mean angle between shoulder-wrist and shoulder-hip vectors (left and right arms) | Equation 2 of main text, then  $\frac{{ARM}_{ANG\_R}+{ARM}_{ANG\_L}}{2}$ |
|  | ELB_ANG_ (deg) | Mean angle between shoulder-elbow and elbow-wrist vectors (left and right arms) | Equation 2 of main text, then  $\frac{{ELB}_{ANG\_R}+{ELB}_{ANG\_L}}{2}$ |
|  | ARM_SI_ (-) | Symmetry Index (SI) between arm angles | Equation 1 of main text |
|  | ELB_SI_ (-) | Symmetry Index (SI) between elbow angles | Equation 1 of main text |
|  | T_ANG_ (deg) | Angle between trunk segment (shoulder centroid to hip centroid) and vertical axes | Equation 2 of main text |
|  | PAUSE (#) | Mean number of pauses |  |
|  | ERROR (#) | Mean number of errors |  |
|  | POINT (#) | Mean number of points |  |
|  | TIME (s) | Mean duration |  |
| SKI | LEG_ANG_ (deg) | Mean angle between shoulder-hip and hip-knee vectors (left and right legs) | Equation 2 of main text, then  $\frac{{LEG}_{ANG\_R}+{LEG}_{ANG\_L}}{2}$ |
|  | KNEE_ANG_ (deg) | Mean angle between hip-knee and knee-ankle vectors (left and right knees) | Equation 2 of main text, then  $\frac{{KNEE}_{ANG\_R}+{KNEE}_{ANG\_L}}{2}$ |
|  | LEG_SI_ (-) | Symmetry Index (SI) between leg angles | Equation 1 of main text |
|  | KNEE_SI_ (-) | Symmetry Index (SI) between knee angles | Equation 1 of main text |
|  | T_ANG_ (deg) | Angle between trunk segment (shoulder centroid to hip centroid) and vertical axes | Equation 2 of main text |
|  | PAUSE (#) | Mean number of pauses |  |
|  | ERROR (#) | Mean number of errors |  |
|  | POINT (#) | Mean number of points |  |
|  | TIME (s) | Mean duration |  |
| PIANO | ARM_ANG_ (deg) | Mean angle between shoulder-wrist and shoulder-hip vectors (only for engaged arm) | Equation 2 of main text |
|  | T_ANG_ (deg) | Angle between trunk segment (shoulder centroid to hip centroid) and vertical axes | Equation 2 of main text |
|  | PAUSE (#) | Mean number of pauses |  |
|  | ERROR (#) | Mean number of errors |  |
|  | KEY_TIME_ (s) | Mean time between consecutive key presses |  |
|  | TIME (s) | Mean duration |  |
| GYM^2^ | ROM_ANG_ (deg) | Mean excursion of arm angle between shoulder-wrist and shoulder-hip vectors (left and right arms) | Equation 2 of main text, then  $\frac{{ROM}_{ANG\_R}+{ROM}_{ANG\_L}}{2}$ |
|  | ARM_VEL_ (m/s) | Mean raising speed of arm (left and right arms) | $\frac{{VEL}_{ARM\_R}+{VEL}_{ARM\_L}}{2}$ |
|  | ARM_SI_ (-) | Symmetry Index (SI) between arm angles | Equation 1 of main text |
|  | VEL_SI_ (-) | Symmetry Index (SI) between arm speeds | Equation 1 of main text |
|  | PPM (#) | Mean raising arm peaks per minute | $\frac{PEAKS}{TIME}*60$ |
|  | MOV_OK_ | Percentage of successful movements (ROM_ANG_ > 60°) |  |
|  | ERROR (#) | Mean number of errors |  |
|  | TIME (s) | Mean duration |  |

The suffix ‘L’ refers to the left side of the body, and the suffix ‘R’ to the right side.

^1^: Computation is indicated only for MFPs. GBMs are recorded by the exergames.

^2^: Computed separately for the three exergame modes (single-arm, alternated, simultaneous). SUCC_MOV_ and ERROR are the average of the left and right arms. In bilateral modes (alternated and simultaneous), PEAKS is the sum of left and right arm peaks.
